# Development and pilot evaluation of a pregnancy-specific mobile health tool: a qualitative investigation of *SmartMoms Canada*

**DOI:** 10.1186/s12911-018-0705-8

**Published:** 2018-11-12

**Authors:** Lyra Halili, Rebecca Liu, Kelly Ann Hutchinson, Kevin Semeniuk, Leanne M. Redman, Kristi B. Adamo

**Affiliations:** 1 School of Human Kinetics, Faculty of Health Sciences, University of Ottawa, Ottawa, 200 Lees Ave, Ottawa, ON K1N 6N5 Canada; 2 Pennington Biomedical Research Center, Baton Rouge, LA USA

**Keywords:** *SmartMoms Canada*, Pregnancy, Mobile health

## Abstract

**Background:**

Mobile technology is ubiquitous. Women of childbearing age have embraced health information technology for pregnancy-related counsel as prenatal care provider communication is increasingly scarce and brief. Pregnant women and new mothers place high value in the use of online sources to support their pregnancy information needs. In Canada, over 300,000 women are pregnant annually, with approximately 60% exceeding evidence-based weight gain recommendations. Mobile health (mHealth) tools, such as mobile applications (app), have the potential to reduce excessive gestational weight gain, offering pregnant women trustworthy guidance, ultimately improving the health outcomes of mothers and infants. Therefore, the primary aim of this study was to implement a qualitative, descriptive research design to assess the receptiveness, functionality, and future prospective of the *SmartMoms Canada* mHealth app.

**Methods:**

Two focus groups (*n* = 13) involving both currently pregnant and recently postpartum women were organized on the same day. Focus groups were transcribed verbatim and thematic analysis was undertaken using manual coding and NVivo software. Participants who took part in the focus groups (n = 13) and those who could not attend (*n* = 4) were asked to complete a Likert-scale survey. All survey responses (*n* = 17) were analyzed using simple tabulation and percentage analysis.

**Results:**

Participants were technologically proficient and interacted with several mHealth tools prior to testing the *SmartMoms Canada* app. Six major themes emerged from thematic analysis: knowledge of pregnancy-specific mHealth services, knowledge and attitudes of weight gain guidelines, weight tracking, strengths of the app, critique and lastly, future suggestions for the app.

**Conclusions:**

Our thematic analysis found that women positively viewed the future potential of our app and offered constructive feedback to improve the next version. Participants sought more personalization and enhanced app interactivity, along with promotion of overall maternal health including nutrition and mental health, in addition to weight tracking.

## Background

Worldwide, over 7 billion mobile cellular subscriptions were reported by the International Telecommunication Union in 2016 [1], rendering mobile technology near-ubiquitous. There is immense potential for mobile technology to provide a platform for initiation of large-scale health promotion interventions aimed at informing personal health parameters. Mobile health (mHealth) technologies, in particular, have already shown significant promise in delivering cost-effective health services in low-to-middle income countries [2], and their use is encouraged by the World Health Organization (WHO) as a means of improving maternal and child health [3, 4].

In Canada, over 300,000 women are pregnant annually [5], with greater than 50% entering pregnancy overweight or obese [6] and nearly 60% exceed Institute of Medicine (IOM) gestational weight gain (GWG) guidelines [7]. Weight gain above guidelines is associated with an intergenerational cycle of obesity [8], affecting the short- and long-term health of both mother and infant. Adverse maternal health outcomes of excessive GWG include pre-eclampsia [9], postpartum weight retention [10], and obesity [11]. Adverse fetal health outcomes include the risk of being born large-for-gestational age [12], downstream metabolic disorder, overweight and obesity [13]. Several epidemiological studies on fetal programming [14–16] have shown that environmental factors in utero (i.e. excessive GWG and maternal obesity) alter one’s susceptibility to downstream disease [17, 18]. Thus, a healthy womb is essential for a healthy pregnancy and optimizing weight gain within guidelines is critical for improving maternal-fetal health outcomes.

Few women report being appropriately counselled on pregnancy weight gain guidelines from their healthcare providers (HCPs) [19, 20]. A recent review found that GWG counselling between healthcare provider (HCP) and patients is often infrequent and inaccurate [Weeks et al., 2018, *Obstetrical & Gynecological Survey,* in press]. Prenatal HCPs also report feeling unequipped to deliver adequate lifestyle counselling [20], including diet and exercise. This is likely a result of these services being resource-intensive, time-consuming, and impractical within the Canadian healthcare system [21]. A need to develop novel, cost-effective, and sustainable approaches to preventing excessive GWG are necessary in the technologically-driven environment of the twenty-first century. Fortunately, pregnant women and new mothers report value in using online sources and mHealth applications (apps) to support their information needs [22], viewing them as central to a healthy lifestyle [23]. The Internet also plays a significant role in women’s pregnancy-related decision-making [24]. Thus, the use of mHealth interventions in prenatal care may increase patient empowerment, leading to efficient care and greater patient satisfaction [25].

Evidently, the development of reliable, evidence-based, health information services through mHealth technologies are necessary to meet the needs of the current and next generation of tech-savvy women. The *SmartMoms Canada* mHealth app, modeled after an earlier US-version [26], is one such app designed to improve weight outcomes during pregnancy. Unlike other pregnancy apps, *SmartMoms Canada*, is predominantly research-based and health-focused. It aims to integrate and maintain healthful lifestyle behaviours such as engaging in physical activity, a healthy diet, managing anxiety, and practicing adequate sleep hygiene. *SmartMoms Canada* offers cost-effective, real-time, personalized support in order to overcome the limitations of standard prenatal care encountered by Canadian women. Herein, the primary objective of this study was to conduct a preliminary exploration of women’s attitudes towards the *SmartMoms Canada* app as a reliable source of information and ability to address lifestyle behaviours related to GWG. Secondly, this study explored women’s receptiveness and critique towards the functionality and feasibility of *SmartMoms Canada* in order to inform the design and methodology for a future multi-centre assessment of the app.

## Methods

### Ethics approval

This study conformed to the regulations set forth by the Declaration of Helsinki. The University of Ottawa, Research Ethics Board (file number: H11–16-17) approved the facilitation of the focus groups. Informed consent was obtained from all interested and eligible participants.

### Study design

A qualitative, descriptive research study design was implemented to assess the utility of the *SmartMoms Canada* mHealth app. A total of two focus groups, occurring on the same day, were organized to evaluate the receptiveness, functionality, and future prospective of the app. Following the completion of the two focus groups, participants were given a self-administered survey composed of only Likert-scale questions. Likert-scale questions were chosen to ensure simplicity of the survey so that participants may understand the questions without the need of guidance from the researchers.

### Study setting, participants, and recruitment

Women who were pregnant or had given birth to a baby within six months of the first scheduled focus group were invited to participate in one of two focus groups at the University of Ottawa, Canada. The city of Ottawa is Canada’s capital city. It is a bilingual city where both official languages, English and French, can be used as desired as a means of communication. It is the fourth largest city in Canada by population (approximately 870,000 or 1,300,000 residents including both the Ottawa-Gatineau region) [27]. Annually, there are between 8000 and 9000 births in Ottawa and a total fertility rate of 1.43 births per woman [28]. Ottawa is an urban center with two major economies formed by the federal government and high technology. The population makeup of Ottawa is predominantly Caucasian [27]. Participants were recruited through a snowball recruitment technique whereby women who participated in other pregnancy-focused studies in our lab, informed their friends and family about the opportunity to participate in upcoming research studies. Social media advertisements were also made on Facebook and Twitter. Recruitment took place from July 2017 to September 2017. All correspondence with participants was completed through a designated *SmartMoms Canada* email operated by Google mail servers at canadasmartmoms@gmail.com. Pregnant and/or postpartum women included in the study were between the ages of 18 to 40 years, fluent in either English or French, had access to Wi-Fi™ and could dedicate at least one week to trial the *SmartMoms Canada* app and accessories. Excluded from the study were women who had a newborn greater than 6 months old by the first scheduled focus group. Women were offered beverages and catered food, were provided with childcare, and were given a $10 gift card for their participation.

### Development of the SmartMoms Canada Mobile health application

The *SmartMoms Canada* app is a new and innovative, evidence-based tool, modelled after an earlier US version [26] that allows mothers-to-be to monitor their GWG and attain a healthy pregnancy weight. This is made possible through stand-alone content with individualized nutrition, physical activity, sleep and other lifestyle recommendations. Women of childbearing age are increasingly tech-savvy and *SmartMoms* may become an important contributor to pregnancy-related mHealth technology. Further, in-person weight management interventions during pregnancy may not be cost-effective and cannot reach a national population. There are several key differences between *SmartMoms Canada* and the earlier US version, called SmartMoms. Our mHealth tool is offered in both official languages of Canada, that is, French and English. Further, our app includes synchronization with a newer ©Fitbit device, the Charge 2 fitness tracker, which offers sleep, exercise, and diet tracking in addition to daily step counts as well as mental health and mindfulness techniques that are complemented by app content. Lastly, there is greater emphasis on physical activity within our app, whereby women are provided with thorough and visually-based exercise guidelines. Therefore, the *SmartMoms Canada* app is a proposed, cost-effective, solution to improve pregnancy outcomes across Canada.

### Procedures and data collection

Participants were asked to take part in two sessions on two separate days. The two sessions were separated by a minimum of 2 weeks or a maximum of 4 weeks and each lasted approximately 90 min. The purpose of the first session was to gather preliminary information on the participating women, introduce them to the study, provide them with the *SmartMoms Canada* app and accompanying technological accessories, and offer detailed guidelines on how to use the study equipment. The purpose of the second session was to have the women participate in one of the two focus groups, scheduled on the same day. The focus groups sought to evaluate women’s perspectives on their interactions with *SmartMoms*. All sessions took place at the Prevention in the Early Years Laboratory located at Lees Campus, University of Ottawa.

During the first session, participants were encouraged to ask questions and to complete a written informed consent document. A preliminary screening form was used to collect age, height, weight, gestational age (if applicable), and age of their newborn (if applicable). The *SmartMoms* app was developed for both Android (Google LLC, Mountainview, CA, USA), and IOS (Apple Inc., Cupertino CA, USA) devices, however, only the Android version was available to individuals participating in the focus groups. The app was downloaded on participants’ personal phones or designated android devices, if necessary. A Fitbit Charge 2 fitness tracker (©Fitbit, Inc. San Francisco, CA, USA) and a BodyTrace scale (©BodyTrace, Inc. New York, NY, USA), were provided and participants were given a minimum of one week to become familiar with the technological accessories.

During the second session, participants arrived at the lab during their scheduled focus group. A 32-item checklist for interviews and focus groups, the Consolidated Criteria for Reporting Qualitative Research (COREQ), was utilized to support the second session [29]. An external expert from the Parriag Group [30] moderated the focus groups in accordance with a moderator’s guide developed specifically for this study. Two members of the study team (LH and KH) were present to take detailed notes. The moderator’s guide was informed by the literature on IOM weight gain recommendations [31], the use of mHealth technologies among pregnant women [32], along with HCP views on mHealth lifestyle interventions and current prenatal care practices and limitations [23]. At the conclusion of each focus group, women completed a 5 to 10 min, self-administered questionnaire that evaluated information on weight tracking practices, diet, and physical activity along with women’s experiences and overall impression of the *SmartMoms Canada* app.

### Data management and analysis

Likert scale responses from the questionnaire were analyzed using simple tabulation. All focus groups were transcribed verbatim. Inductive content analysis [33] and NVivo 11 software for Windows (QSR International Pty Ltd., Doncaster, VIC, AUS) were used to analyze the transcribed data. Multiple complete read-throughs of each transcript were conducted independently by two members of the research team in a four-step process. Firstly, both team members read the combined focus group data independently. Secondly, the team members conducted manual/NVivo open coding of each transcript reading them line-by-line, and adding interpretive notes to the margins beside the text [34, 35] to ensure data immersion was achieved. Thirdly, similar words and codes were collected from the margins onto coding sheets and categorized to generate a coding list. A template analytic style of coding was used to create the coding lists, whereby codes were derived from the data [36]. Memos were made during coding to link observations and to make inferences [37]. Codes representing similar ideas within and across the interviews were clustered into sub-categories/themes. The collection of groups of codes and collapsing of similar or dissimilar groups was done to establish broader higher order categories [38–40]. In the final step, abstraction was used to confirm higher order categories and to establish emergent themes [41]. The two team members conferenced regularly to discuss and resolve any differences in codes and themes at each stage. A third team member who had been present during the focus groups, participated in the final step of the analysis (abstraction) using NVivo to provide further validation of the themes that emerged from the other two authors. Consensus was sought to resolve any theme discrepancies among the three team members.

## Results

### Participant characteristics

A total of 18 (11 pregnant, 7 postpartum), women were interested and eligible to participate in the focus groups. Of these women, 1 could no longer dedicate the time to participate and 17 attended the first appointment and were given the app along with the necessary accessories. Following the first appointment, 4 women were lost to follow-up (3 participants had a scheduling conflict and 1 pregnant participant gave birth on the day of the focus groups). Residents of the City of Ottawa are generally highly educated due to the two main sectors of the economy, the federal government and high technology, along with a large hospital sector. Of the occupational information shared with our research team by the participants, a resident physician in family medicine, a PhD in clinical psychology, an occupational therapist, a midwife, a registered practical nurse, and two employees of the federal government were present during the focus groups. The two focus groups included eight and five participants, respectively, for a total of 13 participants. Of the 13 participants, 11 were found to be recruited through a snowball approach and 2 were recruited through social media advertisements (1 via Facebook and 1 via Twitter). The women were on average 31 ± 3.28 years of age and categorized as having an overweight BMI (27.2 ± 4.16 kg/m^2^) prior to being pregnant. For participants that were pregnant, they were on average five months into their pregnancy and those who had already given birth, were approximately two months postpartum. A summary of participant characteristics is provided in Table 1.

**Table 1:** Participant Characteristics

Characteristic	Mean (SD)
Age (yrs)	31.5 (3.28)
Height (m)	1.62 (0.07)
Pre-pregnancy weight (kg)	66.0 (10.5)
Pre-pregnancy BMI (kg/m^2^)	25.1 (3.55)
Current weight (kg)	71.4 (11.0)
Current BMI (kg/m^2^)	27.2 (4.16)
Gestational age (weeks and days, if pregnant)	20 weeks 2 days
Age of newborn (weeks and days, if postpartum)	9 weeks 2 days

*Note.* A total of 8 women (5 pregnant and 3 new mothers) participated in the first focus group and a total of 5 women (3 pregnant and 2 new mothers) participated in the second focus group. Altogether, a combined total of 13 women (8 pregnant and 5 new mothers) participated in one of the two focus groups. BMI was calculated using the following formula: [weight (kg)] ÷ [height (m)]^2^. Independent sample t-tests (significance level set at alpha = 0.05) demonstrate that pregnant and postpartum women did not differ statistically with respect to age, pre-pregnancy BMI and current BMI. SD refers to standard deviation

### Questionnaire responses

Participant responses on weight tracking practices, diet, and physical activity, along with the women’s experience and overall impression of the *SmartMoms Canada* app are presented in Table 2. The survey response rate was 100%. Of the 17 women who were distributed the survey, 17 completed surveys were given back to the researchers for analysis. Greater than half of the pregnant and postpartum women believed tracking weight gain was ‘somewhat important,’ and maintaining a healthy diet during pregnancy was ‘important.’ It should be noted that approximately one third of the participants believed keeping track of weight gain was ‘important’ and ‘very important’ and nearly half of the women believed being physically active throughout pregnancy was ‘important’ or ‘very important.’ The *SmartMoms Canada* app was found to be ‘somewhat useful’ to the participants (82%). Greater than half of the participants were ‘somewhat likely’ and ‘likely,’ respectively, to use the app throughout pregnancy. Similarly, nearly half of the participants were ‘somewhat likely’ (53%) and ‘likely’ (35%) to recommend the app to someone who was pregnant.

**Table 2:** Attitudes of Pregnant and Postpartum Women who Attended the First Session Toward Weight Gain, Physical Activity, Nutrition, and the Prospective Use of the *SmartMoms Canada* App during Pregnancy

Survey Questions	Pregnant and Postpartum Women’s Responses				
	Not at all important	Somewhat important	Important	Very important	Not sure
How important is/was it for you to keep track of your weight gain during pregnancy?	1 (6%)	10 (59%)	3 (18%)	3 (18%)	0 (0%)
How important is/was it for you to maintain a healthy diet during pregnancy?	0 (0%)	0 (0%)	10 (59%)	7 (41%)	0 (0%)
How important is/was it for you to be physically active throughout your pregnancy?	0 (0%)	2 (12%)	7 (41%)	8 (47%)	0 (0%)
	Not at all useful	Somewhat useful	Useful	Very useful	Not sure
Based on your experience so far, how useful is the SmartMoms Canada app?	0 (0%)	14 (82%)	3 (18%)	0 (0%)	0 (0%)
	Not at all likely	Somewhat likely	Likely	Very likely	Not sure
How likely are you to use the SmartMoms Canada app for your entire pregnancy?	2 (12%)	8 (47%)	6 (35%)	0 (0%)	1 (6%)
How likely are you to recommend the SmartMoms Canada app to someone else who is pregnant?	1 (6%)	9 (53%)	6 (35%)	1 (6%)	0 (0%)

*Note.* A total of 17 survey responses were collected after the completion of both focus groups. This total of 17 women includes the women who participated in the focus groups (*n* = 13) and those who completed the first session of the study but did not attend the focus groups (*n* = 4). Important: all responses are presented as *n* (%), indicating the number of participants who selected this response and percentage of the total in brackets

### Thematic findings

Women were quite receptive to testing the content and functionality of the *SmartMoms Canada app* along with the accompanying technological accessories, for the duration allotted to them prior to attending the focus group. Overall, the app tested well with the sample population and was found to be a useful tool for pregnant women intending to monitor their health, particularly weight and physical activity, throughout pregnancy.

Six major themes and several associated sub-themes emerged from thematic analysis of the focus group transcripts and are presented in Table 3. The first theme was *Strengths of the SmartMoms app*. Participants were in accord that the app provided pregnancy guidance, support, and offered useful pregnancy-related information, particularly as a tool that can contribute to lowering stress levels and distribute informal support that HCPs may not have time to offer. Many women positively viewed the pregnancy-specific exercises and enjoyed the help of the visual provided. These exercises are demonstrated using a short graphics interchange format of a pregnant woman performing a movement in addition to a text description of the exercise. Participants were keen on using the ©Fitbit accessory to track their fitness and notably, their sleep patterns. Women agreed that the information provided in *SmartMoms* about sleep was helpful and provided a greater focus on overall health, in addition to physical activity practices.

**Table 3:** Themes and Subthemes from Focus Groups regarding Content, Functionality, and Future Prospective of *SmartMoms Canada*

Thematic Analysis	Supportive Evidence
1. Strengths of the *SmartMoms* app	
Pregnancy guidance and support	*“I definitely think that for people who haven’t necessarily tried a whole bunch of fitness apps or haven’t tried other pregnancy apps, it’s a fantastic place to start. Because it’s a tool, and when you’re pregnant any tool [is] helpful because it answers your questions and it helps you lower your stress level and having some guidance is wonderful.”* *“I think a lot of people [would] rather turn to apps in pregnancy because it gives them that informal support that their OB or their midwife may not have time to offer them, or they may not feel comfortable asking questions…and it can be a real asset.”* *“I thought it was helpful to have…small pockets of information”*
Pregnancy-specific exercises	*“All the exercises…were great.”* *“I didn’t always read the entire text [associated with exercises], because the visual was a pretty good reminder.”* *“I definitely like that you can see the image of the [pregnant] person”*
Synchronization with ©Fitbit	*“I never had a ©Fitbit before and really being mindful about how much I’m walking…if it had [synched] would’ve been more interesting.”*
Advice on sleep	*“I found them [SmartTips] helpful in general, especially sleep is really interesting, because it’s not something I prioritize or think of at the forefront of health, but it [SmartMoms] kind of explains that when you’re not sleeping enough you’re probably going to eat more, and [this] can be linked to gestational diabetes, so [it] is interesting…I found it was good and made me more well-rounded when it comes to health”*
2. Critique of the *SmartMoms* app	
Design and aesthetic	*“I think part of it is that in a lot of apps you get some visual to cue you to touch things and here because everything was kind of [in] squares…you didn’t have that same visual cues.”* *“I find other apps [have]…really bright colours, there’s things that are standing out, whereas this [SmartMoms] is a little more muted.”*
Interactivity	*“I find something big with [SmartMoms] is there’s no real interactivity…it says, “set goals” but you can’t put a goal in, and it says this is a checklist of things to do but you can’t check off these are things I’ve done.”*
Feedback	*“I think part of it is…it [SmartMoms] kept saying you’re underweight…so [now] what should I be eating that’s healthy?...what’s the next recommendation? I think that little bit of [information on] how to eat more healthy so that I could be in the healthier range would have been… more helpful.”*
Emphasis on weight gain	*“I do find the emphasis on the app, on the weight gain like I would have preferred more on the physical activity [such as] these are things good for your hips at this stage; your body is changing in this way so focus on these stretches.”*
Developmental and technical issues	*“My weight would change 15 lbs depending on where I was taking my weight in my apartment. But it [©BodyTrace scale] was just not synching with the app. So, I did not use it as much as perhaps I should.”* *“I found every time I’d go to settings that’s when it would crash.”*
3. Knowledge of Pregnancy-Specific mHealth Services	
Technologically proficient group that interacted with several mHealth tools	*“I used the BabyCentre app. It doesn’t have any of the health tracking, but it does have a lot of good articles and forums to talk to other moms. [BabyCentre] has updates on where you are in the pregnancy.”* *“…I started using Ovia because a friend of mine who was pregnant last year recommended it to me…and that app [Ovia] in particular helped replace a lot of the heavily been googled forums of “Is this normal?!”*
4. Knowledge and Attitudes of Weight Gain Guidelines	
Strong familiarity of evidence-based guidelines	*“I think we might be a very educated group, but other people aren’t aware of that [IOM guidelines] and they’re still on that perspective that oh I can eat for two”*
Negative views of guidelines	*“I think move away from the numbers and just more education about the concept of healthy weight gain and then healthy eating”*
Positive views of guidelines	*“I think the guidelines are good, but I think it’s important to remember that they’re guidelines.”* *“I think it’s important that people know [the guidelines] but there’s a fine line between having people stressed about [them] and being aware that just because they’re pregnant doesn’t give them free range to gain as much weight as they can”*
5. Weight Tracking	
Feedback on weight from HCP	*“I was weighed at all my appointments, but I think my doctor would have raised it [weight measured] had it been a concern. These appointments are quick – okay, everything is good, okay, bye…do you have any questions and if I don’t have any questions then… I feel like the OB/GYN are really busy and …I found that they were really quick appointments and there wasn’t any concerns then there was nothing raised unless I had questions and in terms of me weighing myself at home, yes I would once in a while mainly just because I was curious.”* *“I was followed by my GP up until 16 weeks and then referred but he never mentioned healthy weight gain or what to expect. He weighed me every time, but I think similarly he would have said something if there was something to worry about.”*
Worry and stress associated with daily weight tracking	*“I don’t like to weigh myself daily because it makes me really anxious and I’m thinking any person with an eating disorder, weighing yourself daily is not good. So maybe just a…reminder once a week might be enough.”*
Weight tracking positively viewed	*“I’ve always weighed myself, once a week or once every two weeks. When I got pregnant I was on the cusp of BMI of overweight, so it was something that I was concerned about, because I don’t want to gain too much. But, having the app…I wanted to test it a lot, I did usually weigh myself every day…but I would go back to weighing myself probably once a week.”*
6. Future Suggestions for *SmartMoms Canada*	
Greater focus on overall maternal health	*“…if you have a baby section maybe have a “what’s going on with your body this week” because for a lot us who are first pregnancies, I thought it’s been a sharp learning curve”* *“I think more focus on the exercise, more focus on the healthy eating, healthy sleeping…[and] water intake. Weight is part of it [health] but…the other things are more important.”* *“…I would have liked more of a healthy mind component as well.”*
Incorporate exercise routines based on time, availability, and trimester	*“Have mini workouts instead of just pick random exercises”* *“Maybe when [SmartMoms] explains our routine, something is already built but that could change throughout the pregnancy. Because what you can do in your first semester is not what you can do in your third semester.”* *“It would be nice to say how much time do I have? And you can have 5 min, 10 min [and then] pick something [or] try this? That sort of thing really engages you.”*
Reassuring feedback and enhanced interactivity	*“Yeah! Some feedback would be good, reassuring.”* *“I’d like if it [SmartMoms] had notifications like “remember to do this today” or exercise plans…okay, I have a plan, I can do this! Instead of, “well, what am I going to do today?”*
Organize app content based on personal health preferences	*“I think it [SmartMoms] could just be visualized based on your health priorities. So, like you said like if you don’t want to see weight maybe it [can] go to the bottom of your dashboard or maybe it’s something you can hide completely. If you’re more focused on making sure you get your greens or your activity, then maybe that could be raised a little higher.”*
Connecting with other pregnant women, using forums, discussion boards, and blogs	*“…on websites there are always discussion boards or a blog you kind of hear from other moms and what they’ve tried….with the app, it’s okay, it’s just what you should do…It’s always nice to get different opinions and what worked a little bit easier for someone and what worked better for someone else.”* *“Maybe it [SmartMoms] could even have a social aspect where you could link with other moms within the radius who have the app.”*

A second theme derived from analysis was *Critique of the SmartMoms app.* Participants were critical, predominantly of the design and aesthetic of the app, such as colour schemes and the layout of the information provided. Women were interested in setting their own goals and interacting with the app in terms of creating their own checklists and to-do lists. Feedback on weight gain was commented upon by the majority of women. The app is designed to provide information on whether guideline-concordant weight gain has been achieved by informing women if they are within or outside the appropriate evidence-based, IOM, weight range. Women requested feedback on how to proceed if they are over or under the weight gain zone. Participants expressed that the app emphasized weight to a greater extent than they would have preferred and requested more emphasis on physical activity. Women also agreed that there were developmental coding errors and technical issues associated with the synchronization of the app and accessories.

The group of women who took part in the focus groups were technologically proficient and informed. From this, a third theme emerged which was the *Knowledge of Pregnancy-Specific mHealth Services.* The women used several mHealth services such as BabyCenter, TheBump, What to Expect, and Ovia health. A fourth theme to arise from analysis was *Knowledge and Attitudes of Weight Gain Guidelines.* Women were knowledgeable of IOM or other evidence-based weight gain guidelines for pregnancy, however, some expressed a negative perspective regarding the guidelines whereas others expressed positive viewpoints. Those who portrayed negative views of the guidelines were critical of the emphasis on a target number as opposed to focusing on healthy eating. Contrarily, those who perceived the guidelines positively viewed them as an important reminder to be mindful of one’s weight and eating habits throughout pregnancy.

Participants were near unanimous on their viewpoints regarding *Weight Tracking*, a fifth theme to emerge from transcript analysis. Most women reported that they often did not receive feedback on weight from their HCP and weight gain guidelines were not discussed. Women made note of the quick nature of prenatal care appointments and emphasized the lack of time that HCPs have to allocate to their patients. Many participants highlighted that daily weight tracking was stress-inducing and would prefer to monitor their weight only once in a while. However, some participants considered themselves weight-centric and positively viewed daily weight tracking.

A final theme to emerge from thematic analysis was *Future Suggestions for SmartMoms Canada.* Participants provided wholesome feedback for the next version of the *SmartMoms Canada* app. Women predominantly viewed the app as a means of tracking weight throughout pregnancy and wanted greater focus on overall maternal health, emphasizing exercise, healthy eating, sleep, and mindfulness. Participants were in consensus that the app should incorporate more feedback in the form of notifications, along with further interactivity that would allow women to input their own goals. Women thoroughly enjoyed the exercises in the app, however, they suggested including short workout routines based on time availability and stage of pregnancy. Participants also wanted to be able to organize the app content based on one’s health preferences and were interested in connecting with other pregnant women who were using the app to discuss pregnancy-specific matters through an embedded discussion board, forum, or blog.

## Discussion

This study explored pregnant and postpartum women’s attitudes towards a novel pregnancy app, *SmartMoms Canada.* Principally, we found that women positively viewed the app with respect to its ability to provide pregnancy guidance, pregnancy-specific exercises, and advice on sleep. From our qualitative analysis, it is clear that pregnant women interact often with mHealth services and seek such tools to provide them with information and informal support that HCPs may not have time to offer. We also found that women who were not weight-centric prior to pregnancy, experience worry and stress with daily weight tracking. Participants highlighted the importance of organizing the app based on their personal health preferences and that emphasis on overall maternal health (i.e. combination of exercise, nutrition, and mindfulness) are included. In addition, women viewed the future prospective of the app to have potential for health promotion during pregnancy and limit excessive GWG given the design, aesthetic, developmental coding errors, and synchronization difficulties are resolved in the upcoming version.

It is of no surprise that women have already taken the lead in utilizing mHealth tools as key sources of information and actively access the Internet for pregnancy-related support, ultimately impacting their decision-making [24, 42]. In accordance with this evidence, the women in the focus groups made use of several pregnancy-specific mHealth tools for guidance. Women embraced the use of the Internet to provide them with efficient information in support of their decision-making that their care providers did not have the resources or time to offer. Future work, however, should focus on evaluating the reliability of information received from websites and apps [25]. Prenatal care providers have voiced concerns over the loss of information control and the health risks associated with exposure to inaccurate information through electronic health (eHealth) and mHealth services [23]. Our app, in particular, is a research-based, non-for-profit, health-focused tool developed by a team of experts in pregnancy lifestyle interventions and maternal-fetal health. Recognizing this, women in the study indicated that they were ‘likely’ and ‘somewhat likely’ to use the *SmartMoms* app throughout pregnancy and to recommend the app to others who are pregnant, thereby encouraging the development of the next version of *SmartMoms Canada*.

On average, the women in this study were categorized as having an overweight BMI prior to entering pregnancy. Our sample demographic is reflective of the Canadian pregnant population. The rate of obesity has significantly increased among Canadian women aged 18 years and older in recent years and is predicted to rise in the near future [43]. Thus, prevention of obesity during pregnancy is of high priority in the Canadian healthcare system. After being presented with the 2009 IOM weight gain guidelines, women in the focus groups recognized the guidelines and generally understood the importance of staying within recommendations. However, some women expressed negative views towards the guidelines in that they are too strict and should focus more on healthy eating and physical activity. Women who view IOM weight gain guidelines in a negative light, should recognize that the lifestyle interventions (i.e. healthy eating, physical activity, and mindfulness) they seek from guidelines are primarily developed to optimize healthier rates of GWG and prevent excess GWG across the entire pregnancy. Since the inception of the IOM guidelines, several studies and systematic reviews have stressed the negative health outcomes for both mother and baby associated with entering pregnancy overweight or obese and gaining above recommendations during pregnancy [44, 45]. Further, a systematic review of 22 research articles found that self-weighing interventions improve weight outcomes, wherein no negative psychological effects are associated with self-weighing at a frequency of daily or weekly [46]. As part of a strategy for gaining weight during pregnancy within the most recent guidelines, the IOM has set detailed recommendations of weight gain per week based on pre-pregnancy BMI [47]. Therefore, a minimum of weekly self-weighing is necessary to ensure positive pregnancy health outcomes. Given our enlightened understanding of women’s thoughts of pregnancy weight gain guidelines from our analysis, the *SmartMoms Canada* app can help inform women of guidelines and further educate them on the importance of guideline adherence in addition to offering lifestyle support such as physical activity, nutrition, and mindfulness advice that women seek.

Healthcare providers have long been sought out to address issues regarding excessive GWG to their patients and provide them with evidence-based information. However, recent work has shown that many are failing to counsel on weight during pregnancy [48]. Importantly, HCPs report that weight is a sensitive topic to discuss with their pregnant patients [49]. A major theme to emerge from our study was the stress, worry, and sensitivity associated with weight tracking. This is in accordance with previous literature, which has found that weight gain may indeed be a sensitive topic for many women during pregnancy [50]. Women in our study reported either a lack of time from HCPs to discuss weight-related concerns unless the women raised the issue or a lack of confidence from HCPs in adequately discussing healthy weight gain. These findings are consistent with the challenges and barriers perceived by HCPs when discussing GWG with patients. mHealth technologies, such as *SmartMoms* may alleviate these challenges thereby providing women with timely GWG counselling. Moreover, *SmartMoms* can provide patients with evidence-based information on weight, healthy eating, exercise, sleep, and mindfulness as an informal support during pregnancy in which women can follow-up with their HCPs.

Thematic analysis found that the future prospective of the *SmartMoms Canada* app was promising, however, several suggestions were offered. Focus group participants emphasised the need for greater interactivity with the app and a desire for enhanced feedback regarding weight, physical activity, nutrition, and mental health. Women suggested the inclusion of exercise routines based on time availability and per trimester, along with being able to organize app content based on personal health priorities. In support of our findings, previous work has found that pregnant patients present with a high demand for medical information and seek web-based pregnancy applications and tools that are personalized, interactive, trustworthy and secure, focused on maternal and mental health, motivational, and easy to use [42, 51]. Given the rapid proliferation of medical information technology, there is an increased need for future work on the health outcomes associated with eHealth and mHealth interventions [25] and a demand for patient groups to become involved in the development and testing of mHealth applications (as presented in this study), as well as to assess the feasibility of embedding such apps in a healthcare setting [42].

### Strengths and limitations

Although the findings of the current study provided important insight on a pregnancy-specific mHealth tool, there are limitations that warrant mention. The relatively small, fairly educated, sample prevents the generalizability of findings to others and with participant recruitment limited to the surrounding region of Ottawa, this may further limit transferability of findings to other settings and participants. Given that women had to contact researchers of their interest to participate, it is possible the study was subject to volunteer bias containing a homogeneous group of women who were health conscious and proficient in mHealth technologies. Despite these limitations, it should be noted that as a pilot evaluation, a variety of constructive feedback was still received. In addition, the pilot feedback provided important insight which will be incorporated into future trials of the *SmartMoms* app, targeting a larger sample size and geographical region. Some strengths to note in the current study include our rigorous procedure for thematic analysis and the use of multiple modalities to analyze the focus group transcripts. Manual coding and NVivo were used for data analysis, and a third team member was added to the analysis procedure to validate codes, categories, and to resolve any discrepancies among the other team members.

### Perspectives

After incorporating qualitative feedback, we postulate that the *SmartMoms Canada* app may be a promising solution to address the gaps in HCP communication related to weight during pregnancy, along with support on physical activity, diet, and sleep. Importantly, *SmartMoms Canada* has been in continuous development since the completion of the focus groups. The integration and synchronization of Wi-Fi™ enabled accessories have been improved upon and will soon operate smoothly in combination with the app. The desire for greater feedback on weight gain expressed by the women in the focus groups, has been met. Women will now be notified on whether they are within or outside the zone of appropriate weight gain for gestational age using a validated mathematical model that takes into account pre-pregnancy weight, current weight gain, and caloric intake. The app then offers interactive suggestions on how to return to or stay within one’s weight zone through advice on nutrition and exercise. The future of mHealth tools and their role in prenatal care will depend on the successful embedding of these evidence-based tools in daily health care routines and encouraging HCPs to integrate such tools in their practice. Such a demand will be fulfilled in the next version of the *SmartMoms Canada* app during a multi-centre, pan-Canadian assessment of the app. Findings from this study and future studies will contribute to offering home pregnancy care, patient empowerment, and revolutionizing prenatal care practices, ultimately contributing to improved maternal-fetal health outcomes.

## Conclusions

Pregnancy is a critical and teachable period during a woman’s lifespan. Women in our study were technologically proficient and comfortable using several pregnancy-specific mHealth tools to track their health. Generally, women found *SmartMoms* to be a useful tool with respect to monitoring GWG, offering exercise tips, and sleep guidance. Women sought greater feedback and interactivity from the app and the need for the next version to be free of developmental coding obstacles so as to ensure an aesthetically pleasing, smoothly functioning tool. Understanding women’s perspectives and attitudes towards a novel, health-focused pregnancy app, as presented here for *SmartMoms Canada*, will help inform future mHealth interventions and encourage healthful behaviours during pregnancy. We believe that such lifestyle interventions can help improve population health by halting downstream health risks associated with weight gain above IOM recommendations, providing women with efficient, informal guidance throughout pregnancy.

## Abbreviations

App (s): Mobile Application (s)
BMI: Body Mass Index
eHealth: Electronic Health
GWG: Gestational Weight Gain
HCP (s): Health Care Provider (s)
IOM: Institute of Medicine
m-Health: Mobile Health
SD: Standard Deviation
WHO: World Health Organization
